# Taming out-of-equilibrium dynamics on interconnected networks

**DOI:** 10.1038/s41467-019-13291-2

**Published:** 2019-11-22

**Authors:** Javier M. Buldú, Federico Pablo-Martí, Jacobo Aguirre

**Affiliations:** 1 Laboratory of Biological Networks, Center for Biomedical Technology (UPM), 28223 Pozuelo de Alarcón, Madrid Spain; 2 Complex Systems Group, Universidad Rey Juan Carlos, 28933 Móstoles, Madrid Spain; 3 Institute of Unmanned Systems and Center for Optical Imagery Analysis and Learning (OPTIMAL), Northwestern Polytechnical University, Xi’an, 710072 China; 4 Grupo Interdisciplinar de Sistemas Complejos (GISC), Madrid, Spain; 5 Complex Systems in Social Sciences Group, Universidad de Alcalá, 28801 Alcalá de Henares, Madrid Spain; 6 Centro Nacional de Biotecnología, CSIC, c/ Darwin 3, 28049 Madrid, Spain

**Keywords:** Applied mathematics, Applied physics, Biological physics, Complex networks

## Abstract

A wide variety of social, biological or technological systems can be described as processes taking place on networked structures in continuous interaction with other networks. We propose here a new methodology to describe, anticipate and manage, in real time, the out-of-equilibrium dynamics of processes that evolve on interconnected networks. This goal is achieved through the full analytical treatment of the phenomenology and its reduction to a two-dimensional flux diagram, allowing us to predict at every time step the dynamical consequences of modifying the links between the different ensembles. Our results are consistent with real data and the methodology can be translated to clustered networks and/or interconnected networks of any size, topology or origin, from the struggle for knowledge on innovation structures to international economic relations or disease spreading on social groups.

## Abstract (web summary)

Network properties can be modified when they interact with other networks, yet most previous results have focused on equilibrium states exclusively. Here the authors introduce a framework to examine the out-of-equilibrium dynamics of evolutionary processes to mimic real-world interconnected networks.

## Introduction

Many real-world networks can be considered as systems with an own identity that connect with other entities forming what is known as a network of networks. For example, the functionality of transportation routes, the Internet, the financial networks or the human brain can only be understood focusing on the connectivity between different networks^1–4^.

Although we are starting to unveil the nature of these complex ensembles, it is well established that some of their properties drastically differ from those of isolated networks. For instance, the robustness of a network can be strongly affected by the interdependency with other networks producing unwanted cascading effects due to node failures^5^. Furthermore, although synchronisability of an isolated network increases when reducing the number of connections between highly connected nodes^6^, networks of networks behave in the opposite way, requiring the creation of new links between hubs to achieve the synchronisation manifold^7^.

The fact that network properties are modified when they interact with other networks opens the possibility of identifying optimal interlinks—connector links from now on—between them. For instance, the importance of a network within a network of networks, measured as the accumulated eigenvector centrality^8^, is strongly dependent on its spectral properties (namely, the largest eigenvalue of its adjacency matrix) and on the specific connector nodes, i.e., nodes with connector links to other networks^9^.

One major limitation of the former analyses is that they have mainly focused on the asymptotic equilibrium state of the system, such as the existence of Nash equilibria^10^, the final state of a cascading process^2^ or the stability of the synchronisation manifold^11–13^. However, many real processes are permanently far from equilibrium, sometimes because the configuration of the system does not allow the existence of equilibrium points, others because changes introduced by external perturbations, such as an exogenous environment, are faster than the time required to reach a permanent regime^14–16^. Networks do not escape from this reality, as the majority of real networked systems are continuously evolving in time^17^.

Under the framework of networks competing for a given resource, a major objective could be to minimise—or maximise—the time to reach equilibrium instead of the final value of a certain outcome. For example, consider two interconnected networks that compete to capture the highest number of moving and/or replicating agents inside them, with the possibility of modifying their connector links. This environment could model companies of the same economical sector competing for clients, innovation or market share^18–20^, research centres or hospitals competing for knowledge, influence or highly qualified professionals^10^, or villages that get in touch to optimise their own financial resilience^21^, to cite a few. In all these cases, the optimal strategy for networks with the highest number of nodes and links is to use peripheral connections to interact with the rest^9,22^. However, such weak connections make the redistribution of moving agents very slow compared with other strategies. This fact leads to a natural question: Why shouldn’t we look for a time-dependent suboptimal connection strategy, which, as compensation, requires much shorter times to reach the equilibrium? Furthermore, what if networks decided to modify their strategy of connection at a certain time to avoid a detrimental equilibrium state?

In this study we describe how dynamical processes on networks of networks arrive to an equilibrium state and the potential guidance of such transient behaviour through the real-time precise management of the links that connect them. In particular, we introduce a new framework based on the spectral properties of the systems under study. The analytical treatment of the problem allows to predict how connector links between networks influence the evolution of the whole system and the consequences of tuning such links in real time. We also propose a diversity of applications describing the potentiality of this new perspective and paths for its further development.

## Results

### Basic concepts and definitions

Our departure point is the study of the out-of-equilibrium dynamics of a process occurring on two networks $A$ and $B$, which interconnect through a limited number of connector links to give rise to a network of (two) networks $T$. The evolution with time of the process is given by

1 $${\bf{n}}(t)={\bf{M}}{\bf{n}}(t-1)={{\bf{M}}}^{t}{\bf{n}}(0)\ ,$$

where ${\bf{M}}$ is a generic transition matrix describing the dynamical process occurring on both networks, ${\bf{n}}(t)$ is the vector that stands for the state of the system at time $t$ and ${\bf{n}}(0)$ is the initial condition (see Supplementary Note 1 for details).

Vector ${\bf{n}}(t)$ represents different magnitudes depending on the nature of the system. It might be people in sociological contexts^23^, organisms or molecules in biological environments^24^, amount of money or goods and services in financial and economic networks^25^, or knowledge in innovation networks^18^, to cite just a few. For simplicity, we will use the generic term population vector for ${\bf{n}}(t)$ and consequently each node $i$ has a population ${n}_{i}(t)$ at time $t$ and each network a population ${P}_{A}(t)$ and ${P}_{B}(t)$ equal to the fraction of the total population that remains on its nodes at each time.

Regarding the asymptotic equilibrium of the system, it is easy to see that ${\bf{n}}(t)\to {{\bf{u}}}_{T,1}$ when time grows, being ${{\bf{u}}}_{T,1}$ the eigenvector associated to the largest eigenvalue of the transition matrix ${\bf{M}}$. Furthermore, ${{\bf{u}}}_{T,1}$ is known to depend strongly on the connector nodes, i.e., those connected through the connector links, as well as on the largest eigenvalues ${\lambda }_{A,1}$ and ${\lambda }_{B,1}$ obtained from the isolated networks $A$ and $B$^9^. For convenience throughout this work, we consider networks $A$ and $B$ such that ${\lambda }_{A,1}\,> \; {\lambda }_{B,1}$. As ${\lambda }_{1}$ grows with the number of links and nodes, we call $A$ the strong network and $B$ the weak one.

The eigenvector associated to the largest eigenvalue of the matrix that represents a network is known as the eigenvector centrality^8^. It has been widely used to measure the topological importance of nodes within a network^26,27^ and has been experimentally proved to be critical in knowledge spreading systems^21,28^. We will name central (C) and peripheral (P) nodes of networks $A$ and $B$ those nodes $i$ with large and small centrality ${({{\bf{u}}}_{1})}_{i}$ respectively, calculated when each network is isolated from the other (and therefore being a constant quantity for each node during the whole process).

Finally, it was shown in ref. ^9^ that connecting two networks through nodes with low centrality (i.e., with a peripheral–peripheral (PP) connection) leads the strong network $A$ to retain the highest possible population, whereas connecting them through central nodes (central–central (CC) connection) benefits the most the weakest network. However, the outcome of both connection strategies was measured at equilibrium, regardless of the time required to achieve it and without considering the ability of networks to modify their interconnections. We will now analyse the importance of studying dynamical systems on networks of networks when they are far from the equilibrium state.

### An illustrative example of why time matters

Let us make use of a recent model on knowledge spreading on innovation networks^18,25,29^. Nodes represent individuals or firms that compete –or collaborate– with the rest for the most valuable knowledge for the production of a certain good or the control of a technique, taking into account that knowledge can only be created through collaboration dynamics and knowledge exchange with the partners. In these works, knowledge exchange is understood as formal or informal Research & Development (R&D) relationships that can be described by bilateral interactions among individuals^30,31^. Furthermore, it has already been shown in many contexts (e.g., the biotechnology industry^19^) that less-linked organisations are the most likely to fail, so connecting to other firms in a networked structure seems to be, in general, a good strategy.

The equation describing the dynamics of knowledge spreading on the network is

2 $${n}_{i}(t)=\sum _{j={\{nn\}}_{i}}{n}_{j}(t-1)\ ,$$

where ${n}_{i}(t)$ is the knowledge accumulated by the individual $i$ at time $t$ and such knowledge can only be transferred from individual $j$ to individual $i$ in one time step if they are connected through a link. ${\{nn\}}_{i}$ represents the nearest neighbours of node $i$. The dynamics can be written in matrix form as

3 $${\bf{n}}(t)={\bf{M}}{\bf{n}}(t-1)={\bf{G}}{\bf{n}}(t-1)\ ,$$

where ${\bf{G}}$ is the adjacency matrix of the network, whose elements are ${G}_{ij}=1$ if nodes $i$ and $j$ are connected and ${G}_{ij}=0$ otherwise.

As we have two networks $A$ and $B$, they compete for the amount of knowledge accumulated in their respective nodes. In particular, they can describe firms or groups of professionals that compete for the most efficient development of a product, social groups such as tribes, villages or regions that compete for the control of agricultural, industrial or military techniques, or any other contexts in which two groups of people interact and exchange knowledge that can make them more competent in the development of a certain task.

Let us suppose both networks are initially disconnected, the strongest starting with a total absence of knowledge and willing to connect to the weakest one through the connector nodes to obtain the maximum possible knowledge at the asymptotic equilibrium, but at the same time minimising the time to such equilibrium. For clarity, in this example we analyse the case in which networks are interconnected through a unique connector link, but as will be shown later generalisation to more links is direct.

**Fig. 1:**
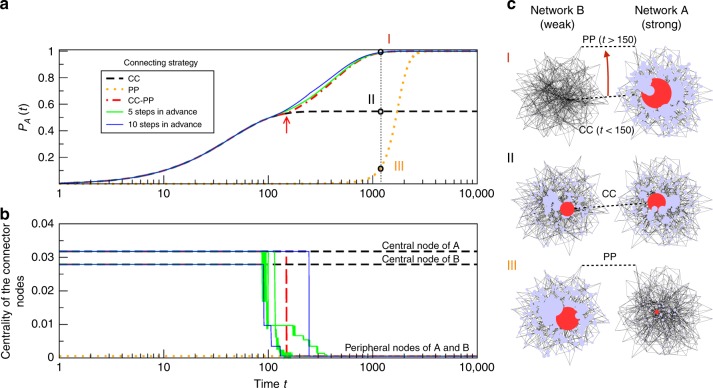
Evolution of a dynamical process on two interconnected networks. They are two different Barabási-Albert (scale-free) networks^36^ of ${N}_{A}={N}_{B}=250$ nodes, ${L}_{A}={L}_{B}=736$ links, ${\lambda }_{A,1}=10.325$ and ${\lambda }_{B,1}=10.253$. We simulate the spreading of knowledge following a model of innovation networks^18,25,29^ where all knowledge is initially placed at the weak network $B$. Networks are initially disconnected and then connect through a single connector link following different connection strategies. **a** Evolution with time of the knowledge accumulated at strong network $A$. The connection strategies are CC (the most central node in $A$ is connected to the most central node in $B$ for all times), PP (the most peripheral node in $A$ is connected to the most peripheral node in $B$ for all times), CC–PP (the networks swap from CC to PP strategy when the CC-asymptotic equilibrium has been reached, indicated by the red arrow at $t=150$) and an exhaustive method choosing the most favourable connection for $A$ from all possible scenarios after 5 and 10 steps. **b** Evolution with time of the connector nodes—those that are connected to the other network—relative to the five different strategies. Each node is represented by its centrality measured in networks $A$ and $B$ in isolation. **c** Plot of the knowledge of both networks at $t=1200$ time steps for the three different strategies indicated by the dotted line in **a**. The radius of the nodes is proportional to their knowledge. Networks’ hubs are indicated in red.

Figure 1 shows several strategies to optimise the total transference of knowledge from a weak network $B$ of ${N}_{B}$ nodes towards a strong network $A$ of ${N}_{A}$ nodes. ${P}_{A}(t)$ accounts for the fraction of knowledge at network $A$ at time $t$, obtained as ${P}_{A}(t)={\sum }_{i=1}^{{N}_{A}}{n}_{i}(t)/{\sum }_{i=1}^{{N}_{A}+{N}_{B}}{n}_{i}(t)$. In consequence, ${P}_{B}(t)=1-{P}_{A}(t)$.

Although most work on economic models are restricted to cliques and stars of different sizes, most real R&D networks are sparse, locally dense and show heterogeneous degree distributions^20,32^. For this reason, and because this heterogeneity is also a frequent property of a large amount of social networks^33–35^, we have used two Barabási-Albert (scale-free) networks for our example^36^. Supplementary Figs. 1 and 2 show equivalent results for collaboration networks of physicians working in two cities in Illinois, USA, and for financial networks of two small villages in India, respectively, showing that the applicability of the phenomenology is independent of the particular properties of the networks under study.

First, we plot the evolution of knowledge in $A$ for two static strategies (i.e., maintaining the connector links along time): connecting networks $A$ and $B$ through nodes with the highest centrality (CC) and connecting through the most peripheral nodes (PP). The former is the fastest strategy, but its final state is very detrimental for the strong network $A$, whereas the latter is the slowest strategy but reaches its optimum asymptotic situation. Next, we implement different dynamic strategies (i.e., modifying if necessary the connector links along time), trying to optimise at the same time the accumulated knowledge and the time to reach it: an exhaustive method in which the connector link maximises the knowledge flux after $n$ steps (for $n=5$ and $10$) exploring all possible combinations and an heuristic method based on choosing a CC strategy (as it leads to fast dynamics far from equilibrium) until the knowledge flux becomes very low, because the CC-asymptotic equilibrium is being reached ($t \sim 150$), and then changing to a PP strategy, which is slower but enables the strong network to obtain its largest possible final amount of knowledge. Figure 1 shows that the exhaustive method is the most effective one, but at a very high computational cost, as, at each time step $t$, we need to calculate the $n$ following steps of the knowledge distribution for all the possible ${N}_{A}\times {N}_{B}$ connections between both networks. On the other hand, the heuristic method based on combining the CC and PP strategies yields a solution that reaches the optimal knowledge for network $A$ and it is only slightly slower than the exhaustive method, but it is several orders of magnitude faster to compute. Finally, note that if the target of the experiment was to hamper the spreading of innovation instead of promoting it, an effective heuristic strategy would be to connect through a PP connection for a long time (minimising the flux of information towards $A$) and, when the remaining information at the weak network is that of the equilibrium when the connection is CC, change drastically to a CC connection to stop the flux.

In summary, the out-of-equilibrium dynamics of the system is strongly dependent on how the connector links are placed between both networks during the evolution of the process. Furthermore, having some intuitions about the phenomenology—such as using in this particular example the heuristic CC–PP strategy—seems to be also of great help. However, how general are these results? Can we solve the problem analytically and obtain general rules to influence the evolution of the system according to our interests? This is the target of the next sections.

### Analytical approach to the phenomenology

Let us cast analytical light to fully describe the phenomenology in a general framework. We focus on two independent networks that are interconnected through a limited number of links, where a generic population (or knowledge, in the example presented above) evolves with time and the connector links can be varied at any time step. We are especially interested in obtaining a theoretical expression for both the population distribution and the population flux—i.e., the fraction of population that goes from one network to the other per time step—at any time, to know precisely the future behaviour of the evolutionary process in function of the properties of both networks, their potential connection strategies and the initial population distribution (see Supplementary Notes 1–8 for the complete analytical analysis of the phenomenology and Supplementary Notes 5 and 6 for the generalisation to directed networks and to systems with more than two networks, respectively).

### Evolution of the population distribution

The evolution with time of a generic process on two interconnected symmetric networks is given by

4 $${\bf{n}}(t)={{\bf{M}}}^{t}{\bf{n}}(0)=\sum _{i=1}^{{N}_{T}}{\lambda }_{T,i}^{t}{\alpha }_{i}{{\bf{u}}}_{T,i}\ ,$$

where ${\bf{n}}(t)$ represents the state of the system at time $t$, **M** is the transition matrix describing the dynamical process, ${N}_{T}={N}_{A}+{N}_{B}$ is the number of nodes in the network of (two) networks $T$ that contains $A$, $B$ and the set of connector links, and ${\alpha }_{i}={\bf{n}}(0)\cdot {{\bf{u}}}_{T,i}$ is the projection of the initial condition on the $i$-eigenvector of matrix ${\bf{M}}$ of network $T$.

We consider networks $A$ and $B$ such that ${\lambda }_{A,1}\;> \;{\lambda }_{B,1}$, with ${{\bf{u}}}_{A,i}$ and ${\lambda }_{A,i}$ the $i$-eigenvector and the corresponding eigenvalue of matrix ${{\bf{M}}}_{{\bf{A}}}$ of network $A$, respectively, and ${{\bf{u}}}_{B,i}$ and ${\lambda }_{B,i}$ the $i$-eigenvector and the corresponding eigenvalue of matrix ${{\bf{M}}}_{{\bf{B}}}$ of network $B$. Let us approximate the initial condition ${\bf{n}}(0)$ to

5 $${\bf{n}}(0)={C}_{A}{{\bf{u}}}_{A,1}+{C}_{B}{{\bf{u}}}_{B,1}\ ,$$

where ${C}_{A}^{2}+{C}_{B}^{2}=1$, and ${{\bf{u}}}_{A,1}$ and ${{\bf{u}}}_{B,1}$ are as mentioned the first eigenvectors associated to networks $A$ and $B$, meaning that initial populations on $A$ and $B$ before interconnecting both networks are already at equilibrium.

We introduce the distribution of population at time $t$ between both networks $x(t)$ as

6 $$x(t)=\frac{{\bf{n}}(t)\cdot {{\bf{u}}}_{B,1}}{{\bf{n}}(t)\cdot {{\bf{u}}}_{A,1}},$$

which is defined between 0 and $\infty$. $x(t)$ tends to 0 when most population is on network $A$ and ${\bf{n}}(t)\to {{\bf{u}}}_{A,1}$ is equal to 1 when the population is equally distributed on $A$ and $B$, and tends to $\infty$ when most population is on network $B$ and ${\bf{n}}(t)\to {{\bf{u}}}_{B,1}$.

Combining Eqs. (4) and (6), and taking into account that applying matrix perturbation theory, we can obtain the first and second eigenvalues and eigenvectors of $T$ as quantities that are only dependent on the eigenvalues and eigenvectors of networks $A$ and $B$ isolated^9^, the weight of the connector links $\epsilon$ and the information of the connector links that connect $A$ and $B$, the evolution with time of the distribution of population $x(t)$ on the network of networks $T$ follows

7 $$x(t)\approx \frac{{K}^{t}L+\frac{\epsilon F}{\Delta \lambda }}{1-{K}^{t}L\frac{\epsilon F}{\Delta \lambda }}+{\mathcal{O}}({\epsilon }^{2})\ ,$$

where

$$K=\left(\frac{{\lambda }_{B,1}\Delta \lambda -{(\epsilon F)}^{2}}{{\lambda }_{A,1}\Delta \lambda +{(\epsilon F)}^{2}}\right),L=\left(\frac{{x}_{0}\Delta \lambda -\epsilon F}{\Delta \lambda +{x}_{0}\epsilon F}\right),$$

$\Delta \lambda ={\lambda }_{A,1}-{\lambda }_{B,1}$, the initial population distribution ${x}_{0}=\frac{{\bf{n}}(0)\cdot {{\bf{u}}}_{B,1}}{{\bf{n}}(0)\cdot {{\bf{u}}}_{A,1}}=\frac{{C}_{B}}{{C}_{A}}$, and

8 $$F={{\bf{u}}}_{A,1}{\bf{P}}{{\bf{u}}}_{B,1}=\sum _{cl}{({{\bf{u}}}_{A,1})}_{i}\cdot {({{\bf{u}}}_{B,1})}_{j}\ ,$$

being $cl$ the set of connector links and ${\bf{P}}$ a matrix whose elements are ${P}_{lm}={P}_{ml}=1$ if nodes $l$ of $A$ and $m$ of $B$ are connected through a connector link, and ${P}_{lm}={P}_{ml}=0$ elsewhere (see Supplementary Note 2 for the whole analytical calculation). Therefore, $F$ represents the sum of the products of the eigenvector centralities of all connector nodes measured prior to interconnecting $A$ to $B$, and for this reason we will name connection strength to $\epsilon F$ and normalised connection strength to $\epsilon F/\Delta \lambda$. $F$ can include as many connector links as desired—as far as the nature of the system is that of two interconnected networks—and such connector links might connect any nodes in networks $A$ and $B$. It is noteworthy that PP connections between networks lead to low values of $F$, whereas CC connections (or a very large number of PP connections) lead to large values of $F$.

Interestingly, the expression for $x(t)$ only depends on the first eigenvalues of the isolated networks ${\lambda }_{A,1}$ and ${\lambda }_{B,1}$, the centrality of their connector nodes (through $F$, which depends on the first eigenvectors ${{\bf{u}}}_{A,1}$ and ${{\bf{u}}}_{B,1}$), the value of the weight of the connector links $\epsilon$ and the initial condition (through ${x}_{0}$).

In case we used a generic initial condition ${\bf{n}}(0)$ different to Eq. (5), the analytic expression becomes more troublesome during the beginning of the process, but in practice, after some steps, the population would spread over both networks and Eq. (7) would be fully applicable (Supplementary Note 4).

In summary, Eq. (7) describes the dynamics of the system for all times and can be used to obtain how much population will be at each network at any time and how the system would evolve if we changed the connector links following any connection strategy.

### The population flux and the asymptotic equilibria

The population flux $\dot{x}(t)$ is a measure of how fast the population of network $A$ spreads towards network $B$ at time $t$. It is an especially important information, as it allows the networks to choose at each step of the process the optimum strategy to maximise or minimise such flow, depending on their particular interests. Making use of the expression for $x(t)$ at Eq. (7) we obtain

9 $$\dot{x}(t)=\frac{dx(t)}{dt}\approx {K}^{t}{\mathrm{ln}}\left(K\right)L\frac{1+{\left(\frac{\epsilon F}{\Delta \lambda }\right)}^{2}}{{\left(1-{K}^{t}L\frac{\epsilon F}{\Delta \lambda }\right)}^{2}}+{\mathcal{O}}({\epsilon }^{2})\ ,$$

where $K$, $L$, $\Delta \lambda$, $\epsilon$ and $F$ are as in Eq. (7) (see Supplementary Note 3 for the analytical calculation).

High values of $| \dot{x}(t)|$ indicate fast changes in the population distribution, whereas $\dot{x}(t)=0$ means that the system has attained an equilibrium state that is obtained from Eq. (7):

10 $$x(t\to \infty )\to {x}_{{\rm{eq}}}=\frac{\epsilon F}{{\lambda }_{A,1}-{\lambda }_{B,1}}\ ,$$

(see Supplementary Note 7 for details). When both networks are connected through peripheral nodes (PP strategy) and therefore $\epsilon F\approx 0$, ${x}_{{\rm{eq}}}=0$ and the equilibrium distribution of population fills the strong network ($A$) and almost coincides with ${{\bf{u}}}_{A,1}$. On the other hand, CC strategies—i.e. large values of $\epsilon F$—push the population as much as possible towards the weak network ($B$), in full agreement with Fig. 1 and ref. ^9^.

Furthermore, from Eq. (9) we obtain that when the initial population distribution ${x}_{0}$ is larger than that of the equilibrium state, i.e., ${x}_{0}\;> \; {x}_{{\rm{eq}}}$, the flux $\dot{x}(t)\;<\; 0$ for all times, whereas ${x}_{0}\;<\; {x}_{{\rm{eq}}}$ implies $\dot{x}(t)\;> \; 0$ for all times. In other words, if the connecting strategy (i.e., $\epsilon F$) between both networks is not changed during the process, the population will flow monotonically from $B$ to $A$ until the equilibrium is reached if ${x}_{0}\;> \; {x}_{{\rm{eq}}}$ and from $A$ to $B$ if ${x}_{0}\;<\; {x}_{{\rm{eq}}}$.

**Fig. 2:**
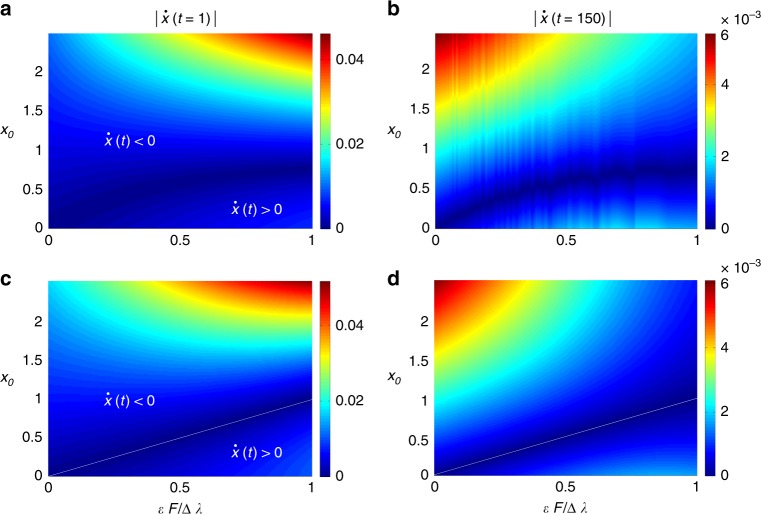
Comparison between the numerical and analytical calculations of the population flux. Population flux $\dot{x}(t)$ (in absolute value, for clarity) as a function of the normalised connection strength $\epsilon F/\Delta \lambda$ and the initial population distribution ${x}_{0}$ for two connected networks out of equilibrium. **a**, **b** Numerical calculation of $\dot{x}(t)$ for two scale-free networks of $N=250$ nodes (see caption of Fig. 1 for details), for $t=1$ (**a**) and $t=150$ (**b**). **c**, **d** Analytical approximation to $\dot{x}(t)$ for the same situations. These plots show that Eq. (9) is in quantitative agreement with the numerical results, despite only depending on the first eigenvalues of networks $A$ and $B$, and the centrality of the connector nodes. In the numerical simulations (**a**–**b**), the connection strength $\epsilon F$ has been modified by both changing the connector links (i.e., $F$) and the weight of the connector links (i.e., $\epsilon$). This way, we have gradually swept $\epsilon F/\Delta \lambda$ from 0 to 1.

Figure 2 shows the absolute value of the population flux $| \dot{x}(t)|$ as a function of the normalised connection strength $\epsilon F/\Delta \lambda$ and the initial population distribution ${x}_{0}$, for $t=1$ and $t=150$, and for two connected networks out of equilibrium calculated numerically (a,b) with the scale-free networks used in Fig. 1, and analytically (c,d) through Eq. (9). We can observe the full agreement between both plots for low and high values of the time $t$, even when the analytical approximation ignores the influence of all ${N}_{A}+{N}_{B}-2$ eigenvalues and eigenvectors of order larger than 2. Importantly, the white lines of Fig. 2(c) and (d) correspond to the equilibrium states ${x}_{0}={x}_{{\rm{eq}}}$. It is noteworthy that the population flux beyond the equilibrium line pushes the population towards network $A$, whereas the flux tends towards network $B$ if we are under that boundary line, as predicted by the analytical treatment of the phenomenology. Also, during the first steps of the process the largest flux is found for CC connections far away from the equilibrium (top-right corner of a and c). However, for large times, networks coupled with PP connections, which are still far from equilibrium, show the fastest dynamics (top-left corner of b and d).

### The population flux diagram and applicability of the framework

**Fig. 3:**
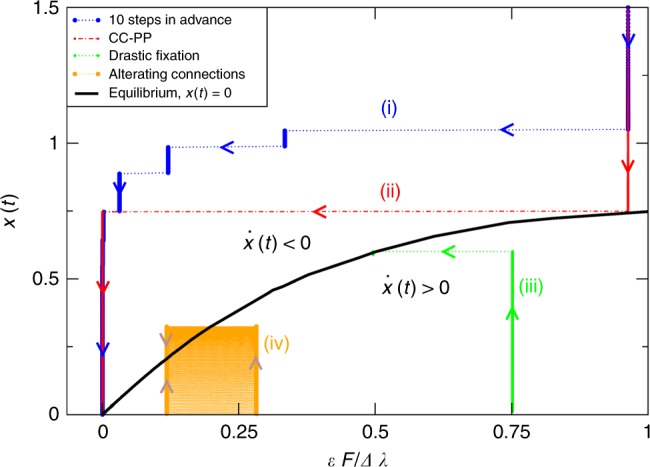
Population flux diagram. We propose this tool for the analysis of generic evolutionary processes on two connected networks out of the equilibrium. It is noteworthy that the normalised connection strength plotted in the X-axis depends on the connector links, whereas the Y-axis represents the population distribution $x(t)$. In consequence, the evolution of a process towards the equilibrium, where changes in the connector links between networks are permitted at any time, becomes a path in the population flux diagram composed of vertical and horizontal lines. For clarity, four different trajectories are plotted for two connected scale-free networks (see caption of Fig. 1 for networks details).

We can use the results obtained above to create a population flux diagram to be used as a general framework for the study of processes over generic interconnected networks out of equilibrium. Specifically, we make use of the following: first, the equilibrium curve obtained through $\dot{x}(t)=0$, which depends on the internal topology of the networks (i.e., their maximum eigenvalues) and the connector links between both networks, but is independent of the initial population distribution ${x}_{0}$; and second, $\dot{x}(t)\;<\; 0$ $\forall t$ when ${x}_{0}\;> \; {x}_{{\rm{eq}}}$ and $\dot{x}(t)\;> \; 0$ $\forall t$ when ${x}_{0}\;> \; {x}_{{\rm{eq}}}$. Therefore, orbits in a diagram $[\epsilon F/\Delta \lambda ,x(t)]$ (as shown in Fig. 3) will move at every step a vertical distance $\dot{x}(t)$ given by Eq. (9) towards the equilibrium boundary, while changing the connector links will displace the orbit horizontally at any time. For example, changing the connector links conveniently permits the orbit to cross the equilibrium curve horizontally, enabling the change of sign of the flux.

This way, we obtain a diagram that allows representing full trajectories from any initial condition to any final equilibrium state permitting to choose the connector links at each time step. If we face the phenomenology as a game^10^, any kind of competition rules are included in this framework, e.g., a unique network seeking for acquiring the largest population in the shortest time, a competition between networks in which competitors alternate in choosing the connector links, etc.

In Fig. 3, we have plotted several sample orbits to show the versatility of this framework. First, most of the population is placed initially on the weak network $B$ (${x}_{0}=2.5$) and the target is to move it to network $A$ as fast as possible. This can be done through the exhaustive method presented in Fig. 1 where at each step the system chooses the connections that maximise the flux of population after ten steps and gradual changes of strategy from CC to CP, and finally to PP connections are shown (trajectory (i)); but also through the heuristic method in which the networks are connected through a CC connection until the system gets close to the equilibrium state and then changes to a PP connection (trajectory (ii)). Second, the population is initially placed on network $A$ (${x}_{0}=0$). We choose an intermediate connection that pushes the population towards network $B$ and when $x(t)$ reaches a certain value ($x(t)=0.6$ in this case) the connections and/or their weights are changed to drastically stop the flux and maintain the population in that situation indefinitely (trajectory (iii)). Finally, to exemplify the applicability of the framework to mathematical games where each network/competitor follows in turns a connection strategy in order to reach a favourable final situation, in trajectory (iv) we study the simplest case of the alternation of two different strategies. This methodology shows that the final equilibrium of this situation is equivalent to that of applying both strategies simultaneously but weighting the connector links with half of their weight (see Supplementary Note 8 for an analytical proof).

Besides the generic examples shown in Fig. 3, the analysis of a real case might be of use to show the potentiality of our framework. Let us focus on the Organisation for Economic Co-operation and Development (OECD) Inter-Country Input-Output database^37^ and study the evolution of the economic networks of the 36 OECD countries from 2005 to 2015 through the analysis of time series associated to finite Markov chains^38,39^. Concepts such as networked economy and its relation with complexity have increased in importance recently, in particular after the financial crisis of 2008^38,40–42^, although Leontief^43,44^ presented its famous input–output model almost a century ago. In these networks, and so in ours, each node is a segment of the economy of a country and the links, which are weighted and directed, represent the monetary flows of goods and services between such nodes. In the Leontief theory, the input–output tables are used as systems of linear equations and their solution is the reached equilibrium after a whole year. Following this idea, our model follows Eq. (1) where **M** is the transition matrix that models the evolution of the economic network and we suppose that every year a different equilibrium is reached. If ${\bf{L}}$ is the input–output table obtained from ref. ^37^, ${M}_{ij}={L}_{ji}/{\sum }_{i}{L}_{ji}$, and therefore each element ${M}_{ij}$ is the fraction of economic sector $i$ that flows to economic sector $j$. This makes **M** a stochastic matrix and the process a Markov chain. As this is a closed model, the system can be interpreted as one in which there is no demand, or on the contrary as one in which the demand is considered as a sector that consumes the totality of its own output^45^. The elements of population ${\bf{n}}(t)$ represent the relative amount of money (or goods and services) in each economic sector at time $t$, and their long-term value given by the elements of eigenvector ${{\bf{u}}}_{T,1}$ can be interpreted as a quantity that is similar to the annual gross domestic product shares^38^. The population distribution $x(t)$ of a country, therefore, yields how much relative amount of money has accumulated in comparison with the whole OECD.

**Fig. 4:**
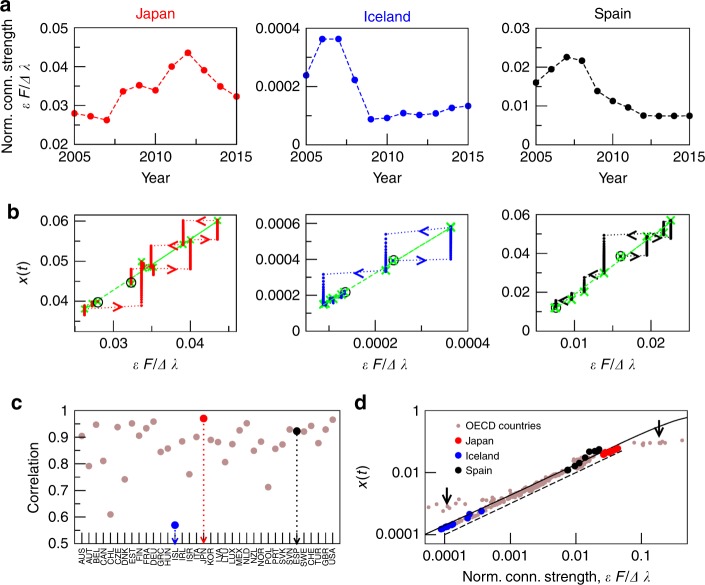
Analysis of the evolution of the OECD economic network from 2005 to 2015. Data obtained from ref. ^37^. **a** Evolution with time of the normalised connection strength for Japan, Iceland and Spain vs. the rest of the OECD. **b** Population flux diagram for the analysis of the economic behaviour of Japan, Iceland and Spain in their interaction with the rest of the OECD. Crosses represent the equilibria and circles are the initial and final equilibria. **c** Correlation between the normalised connection strength for every country vs. the rest of the OECD in a year and its precedent, averaged along the 11-years period. **d** Equilibria for every year (2005–2015) and every country in the OECD in the population flux diagram—USA and Luxembourg marked with arrows. The black line represents the equilibria of the two scale-free networks studied throughout the paper (already plotted in Fig. 3) and the dashed line represents the analytical approximation for such equilibria (${x}_{{\rm{eq}}}=({{\bf{u}}}_{B,1}^{L}{\bf{P}}{{\bf{u}}}_{A,1})/({\lambda }_{A,1}-{\lambda }_{B,1})$).

Figure 4 represents a numerical study of this dataset from the optics of the methodology presented here, focusing on the economic behaviour of the different countries after the crisis of 2008. There are 36 countries and 36 sectors per country, 1296 nodes in total, and $i$ and $j$ can belong to the same or different country. As we are facing a directed network and each link has a different weight, the normalised connection strength between two networks $A$ and $B$ (being $A$ the strong one) is given by $({{\bf{u}}}_{B,1}^{L}{\bf{P}}{{\bf{u}}}_{A,1})/({\lambda }_{A,1}-{\lambda }_{B,1})$. It is noteworthy that only the connector links that go from the strong to the weak network are relevant (see Supplementary Note 5 for details). This quantity can be calculated between each pair of countries (see Supplementary Movie 1), but also between a country and the rest of the OECD, the latter analysed as a unique network and where the connection strength represents the inputs received by a country from the rest of the OECD.

Figure 4a plots the evolution of the normalised connection strength for Japan, Iceland and Spain with the rest of the OECD in the 2005–2015 period. The population flux diagram for the analysis of their economic behaviour is plotted in Fig. 4b. In Fig. 4c, the correlation between the connection strength in a year and its precedent, averaged along the 11-years period and for all OECD countries, is shown, and in Fig. 4d we plot the equilibria for every year and every country in the population flux diagram. Our new point of view shows that Japan is the country with the largest correlation, which means that its economic relation with the rest of the OECD has remained similar during the whole period. In fact, it is almost the only country whose connectivity with the OECD grew after the 2008 crisis, yielding an increase in its relative amount of goods and services (${x}_{{\rm{Japan}}}(2015)\, > \, {x}_{{\rm{Japan}}}(2005)$). Iceland, the country with the lowest correlation, behaved very differently. Its refusal to save the bank system after the crisis is well known. The small country opted for a change in its productive model, modifying in consequence drastically the connections with the rest of the world (see Supplementary Movie 1). This did not prevent Icelanders falling sharply in their connections and their relative amount of goods and services (${x}_{{\rm{Iceland}}}(t)$) after the crisis, but permitted them to recover slowly but in a stable manner. Furthermore, we have followed an intermediate case, Spain. It suffered a drastic decrease in 2008 mainly due to its brick-based economy. The strong impact of the financial adjustment and the difficulties to change the productive model in a relatively large and populated country (the correlation of Spain data is large, see Fig. 4c) prevented it from recovering so fast and up to 2015 its economic situation was still far behind that of 2005 (${x}_{{\rm{Spain}}}(2015)\,<<\, {x}_{{\rm{Spain}}}(2005)$).

Finally, Fig. 4d shows that the 11 equilibrium points of each OECD country, with the exception of the boundary cases (USA and Luxembourg, the largest and lowest maximum eigenvalues of the OECD), collapse on the curve of equilibria plotted in Fig. 3 for the two scale-free networks studied throughout the paper. This fact reinforces the generality of the methodology and the analytical results here presented (plotted in dashed line), and it shows that the evolutionary dynamics that take place on a network of networks mainly depends on the connector links and the largest eigenvalues of the networks, but not on their topology or the nature of the process.

## Discussion

In this study we have shown that an adequate alteration of the connector links between two interconnected networks allows for the guidance of the dynamics of the system from any initial condition to any desired final state, ruling its evolution according to our necessities. We present a full analytical treatment of the process and, in order to simplify the management of the system, we introduce the population flux diagram, a two-dimensional structure that contains the evolution of the population along time comprising all possible initial conditions and connection strategies. Our methodology is applicable to directed and non-directed networks of any size or topology, as long as the dynamical evolution of the system can be modelled through a transition matrix. Furthermore, systems with more than two networks can also be studied from this novel perspective, although the analysis becomes more complex.

For the sake of clarity, we first introduced a simple but illustrative example based on a model that describes the spreading of knowledge on a social network^18,25,29^ and verifies in its simplest version ${\bf{M}}={\bf{G}}$, i.e., the transition and the adjacency matrices coincide. However, our results can be applied to any dynamical process described as ${\bf{n}}(t)={{\bf{M}}}^{t}{\bf{n}}(0)$, being ${\bf{n}}(t)$ the state vector of a certain variable at time $t$ at each node of the network. As a guiding example, we have studied the economic networks of the 36 OECD countries from 2005 to 2015. Although this is just a partial study of the data, whose full analysis is a promising line of research but whose extension goes beyond the scope of this work, our methodology faces the economic behaviour of the different nations from a novel perspective that could be of help to develop active economic policies depending on the economic and financial situation of the country under study. Furthermore, we have devoted Supplementary Note 9 to facilitate the potential application of our methodology to other complex systems, such as mutation-selection evolutionary processes^22,46^, the control and optimisation of the growth of species on fragmented habitats^47^, the description of disease spreading on social environments^8^, and the potential analysis of a dataset that describes the evolution between 1988 and 1999 of the inter-organisational collaboration network in biotechnology^20^.

A different future line of research would be to extend this methodology to analyse the dynamics of processes on a single network where the links can be modified to tune the speed towards the equilibrium or to retain the population within a certain region of target nodes. Finally, we believe that the current results may inspire further studies about how clustered networks, networks of networks or multilayer networks^48^ behave out of equilibrium where other variables such as robustness or vulnerability are the ones to be maximised (or minimised). As long as we find networks where groups of nodes can be assigned to have a common property (i.e., belonging to the same layer, cluster or sub-network), the methodology proposed in this study could be adapted to describe and manage the out-of-equilibrium evolution of the whole system.

## Supplementary information

Supplementary Information

Description of Additional Supplementary Files

Supplementary Movie 1

## Data Availability

All relevant data are available from the authors upon request.

## Code availability

All relevant codes are available from the authors upon request.
