# Supplementary material for: Taming out-of-equilibrium dynamics on interconnected networks
Source: Nat Commun. 2019 Nov 22;10:5314. doi: 10.1038/s41467-019-13291-2 (PMC6874616; doi:10.1038/s41467-019-13291-2)
Supplement: Supplementary file 1 — Supplementary Information [file 41467_2019_13291_MOESM1_ESM.pdf]

SUPPLEMENTARY INFORMATION:

Taming out-of-equilibrium dynamics on interconnected networks

Jacobo Aguirre, Federico Pablo-Martí and Javier M. Buldú.

Contents

Supplementary Figures 2
Supplementary Note 1. Dynamical equations of evolutionary processes on networks 4
Supplementary Note 2. Evolution of the population distribution 4
Supplementary Note 3. The population flux and the asymptotic equilibria 6
Supplementary Note 4. Population distribution and flux for arbitrary initial conditions 7
Supplementary Note 5. Generalisation to directed networks 8
Supplementary Note 6. Generalisation to more than 2 networks 8
Supplementary Note 7. Equilibrium configurations 10
Supplementary Note 8. Alternating strategies 10
Supplementary Note 9. Applicability of the framework 11

Supplementary Figures

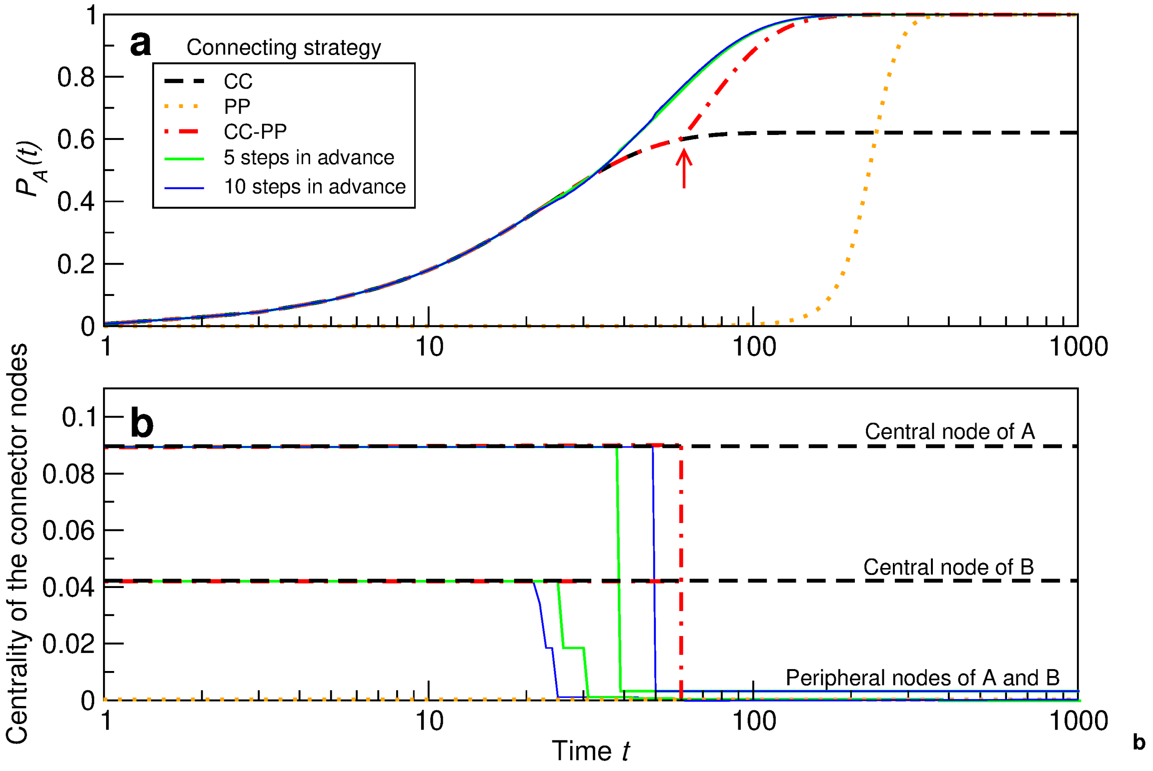

Supplementary Figure 1. Evolution of a dynamical process on two collaboration networks of physicians working in Bloomington (A) and Peoria (B) (Illinois, US). The networks' properties are $N_A = 44$ nodes, $N_B = 113$ nodes, $L_A = 99$, $L_B = 246$ links, $\lambda_{A,1} = 6.4144$ and $\lambda_{B,1} = 6.1566$, from [1, 2]. We simulate the spreading of knowledge following a model of innovation networks [3–5] where all knowledge is initially placed at the weak network B, the networks are initially disconnected and then connect through a single connector link following different connection strategies. (a) Evolution with time of the knowledge accumulated at strong network A. The connection strategies are CC (the most central node in A is connected to the most central node in B for all times), PP (the most peripheral node in A is connected to the most peripheral node in B for all times), CC-PP (the networks swap from CC to PP strategy when the CC-asymptotic equilibrium has been reached, indicated by the red arrow at $t = 60$), and an exhaustive method choosing the most favourable connection for A from all possible scenarios after 5 and 10 steps.

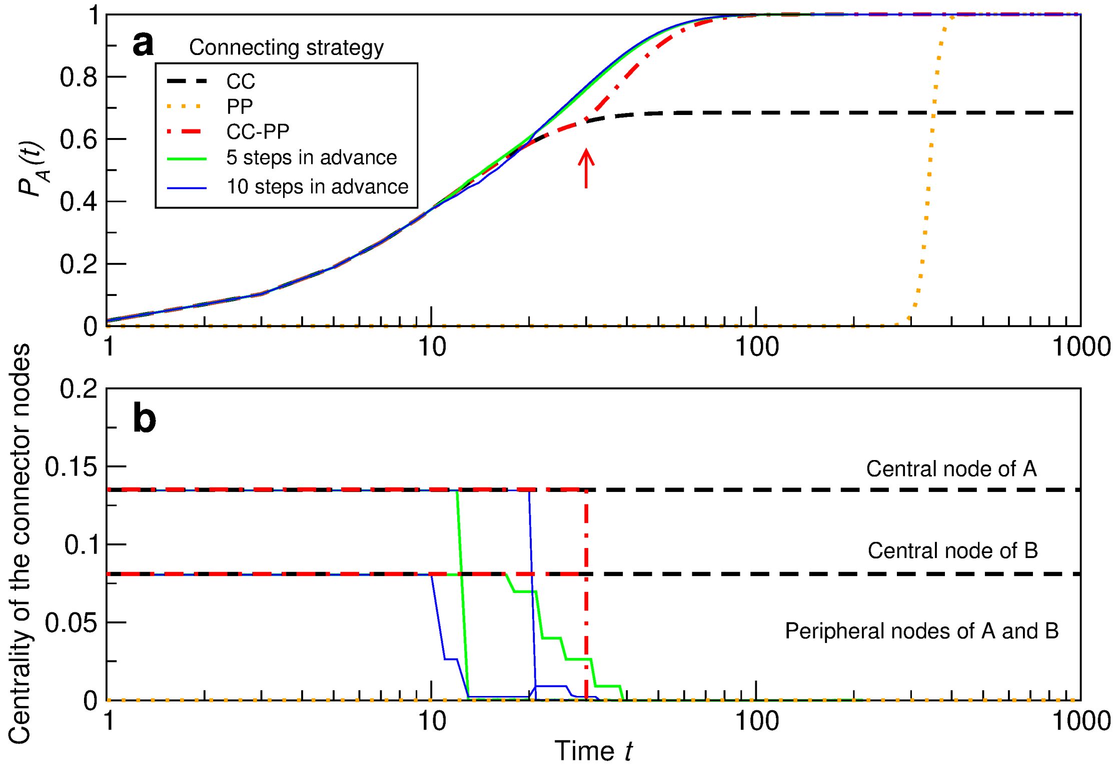

Supplementary Figure 2. Evolution of a dynamical process on the financial networks of two Indian villages. The networks' properties are $N_A = 191$, $N_B = 138$ nodes, $L_A = 242$ and $L_B = 190$ links, $\lambda_{A,1} = 5.0785$ and $\lambda_{B,1} = 4.6961$, networks 60 and 44 of [6]. We simulate the spreading of knowledge following a model of innovation networks [3–5] where all knowledge is initially placed at the weak network B, the networks are initially disconnected and then connect through a single connector link following different connection strategies. (a) Evolution with time of the knowledge accumulated at strong network A. The connection strategies are CC (the most central node in A is connected to the most central node in B for all times), PP (the most peripheral node in A is connected to the most peripheral node in B for all times), CC-PP (the networks swap from CC to PP strategy when the CC-asymptotic equilibrium has been reached, indicated by the red arrow at $t = 30$), and an exhaustive method choosing the most favourable connection for A from all possible scenarios after 5 and 10 steps.

Supplementary Note 1. Dynamical equations of evolutionary processes on networks

The phenomenology here analysed can be applied to evolutionary processes described as

$$\mathbf{n}(t) = \mathbf{M}\mathbf{n}(t-1) = \mathbf{M}^t\mathbf{n}(0) \quad (1)$$

where $\mathbf{M}$ is the transition matrix, $\mathbf{n}(t)$ is the vector that stands for the state of the system at time t and $\mathbf{n}(0)$ is the initial condition.

Let us call λ_i the set of eigenvalues of $\mathbf{M}$, with $\lambda_i \geq \lambda_{i+1}$, and $\mathbf{u}_i$ the corresponding eigenvectors. Its eigenvectors verify $\mathbf{u}_i \cdot \mathbf{u}_j = 0$, $\forall i \neq j$ and $|\mathbf{u}_i| = 1$, $\forall i$. Since $\mathbf{M}$ is a primitive matrix, the Perron-Frobenius theorem assures that the largest eigenvalue of $\mathbf{M}$ is positive, $\lambda_1 > |\lambda_i|$, $\forall\ i > 1$, and its associated eigenvector is positive (i.e., $(\mathbf{u}_1)_i > 0$, $\forall\ i$). The dynamics of the system, Eq. (1), can be thus written as

$$\mathbf{n}(t) = \mathbf{M}^t\mathbf{n}(0) = \sum_{i=1}^{N_T} \lambda_i^t \alpha_i \mathbf{u}_i\,, \quad (2)$$

where $\alpha_i = \mathbf{n}(0) \cdot \mathbf{u}_i$. Furthermore, as $\lambda_1 > |\lambda_i|$, $\forall i > 1$, the asymptotic state of the population is proportional to the eigenvector that corresponds to the largest eigenvalue, $\mathbf{u}_1$, and the largest eigenvalue λ_1 yields the growth rate of the population at equilibrium. If we normalise the population $\mathbf{n}(t)$ such that $|\mathbf{n}(t)| = 1$ after each generation, $\mathbf{n}(t) \to \mathbf{u}_1$ when $t \to \infty$.

A note on the mathematical notation used in this paper: when the system represents two networks A and B interconnected through a set of connector links, the first N_A elements of $\mathbf{n}(t)$ correspond to the nodes of network A and the N_B remaining elements correspond to the nodes of network B. Therefore, the length of $\mathbf{u}_{T,i}$, $\mathbf{u}_{A,i}$ and $\mathbf{u}_{B,i}$ is $N_A + N_B$. The first N_A elements of $\mathbf{u}_{A,i}$ are equal to the i-eigenvector of network A isolated and their last N_B elements are equal to zero, while the first N_A elements of $\mathbf{u}_{B,i}$ are equal to zero and their last N_B elements are equal to the i-eigenvector of network B isolated.

Supplementary Note 2. Evolution of the population distribution

The evolution with time of a generic process on two interconnected non-directed networks is given by

$$\mathbf{n}(t) = \mathbf{M}^t\mathbf{n}(0) = \sum_{i=1}^{N_T} \lambda_{T,i}^t \alpha_i \mathbf{u}_{T,i}\,, \quad (3)$$

where $\mathbf{n}(t)$ represents the state of the system at time t, $\mathbf{M}$ is the symmetric transition matrix describing the dynamical process, $N_T = N_A + N_B$ is the number of nodes in the network of (two) networks T that contains A, B and the set of connector links, and $\alpha_i = \mathbf{n}(0) \cdot \mathbf{u}_{T,i}$ is the projection of the initial condition on the i-eigenvector of matrix $\mathbf{M}$ of network T (see Supplementary Notes 5 and 6 for the generalisation to directed networks and more than two networks respectively). We consider networks A and B such that $\lambda_{A,1} > \lambda_{B,1}$, with $\mathbf{u}_{A,i}$ and $\lambda_{A,i}$ the i-eigenvector and the corresponding eigenvalue of matrix $\mathbf{M_A}$ of network A respectively, and $\mathbf{u}_{B,i}$ and $\lambda_{B,i}$ the i-eigenvector and the corresponding eigenvalue of matrix $\mathbf{M_B}$ of network B. Note that $\mathbf{M}$ consists of four blocks of elements: two diagonal blocks $\mathbf{M_A}$ and $\mathbf{M_B}$ that represent the internal interactions between nodes in networks A and B respectively, and two off-diagonal blocks that will be equal to zero if networks A and B are totally disconnected, and will show the connector links from A to B and vice versa if they eventually connect. Both internal and connector links are of the same nature, being ϵ the weight of the connector links.

Applying matrix perturbation theory (assuming small ϵ) we obtain that the first and second eigenvalues and eigenvectors of T can be expressed as quantities that are only dependent on the isolated networks A and B [8]:

$$\mathbf{u}_{T,1} = \mathbf{u}_{A,1} + \epsilon \sum_{k=1}^{N_B} a_k \mathbf{u}_{B,k} + \epsilon^2 \sum_{j=2}^{N_A} \sum_{k=1}^{N_B} \frac{a_k(\mathbf{u}_{A,j}\mathbf{P}\mathbf{u}_{B,k})}{\lambda_{A,1} - \lambda_{A,j}} \mathbf{u}_{A,j} + o(\epsilon^3)\,, \tag{4}$$

$$\mathbf{u}_{T,2} = \mathbf{u}_{B,1} + \epsilon \sum_{k=1}^{N_A} b_k \mathbf{u}_{A,k} + \epsilon^2 \sum_{j=2}^{N_B} \sum_{k=1}^{N_A} \frac{b_k(\mathbf{u}_{B,j}\mathbf{P}\mathbf{u}_{A,k})}{\lambda_{B,1} - \lambda_{B,j}} \mathbf{u}_{B,j} + o(\epsilon^3)\,, \tag{5}$$

$$\lambda_{T,1} = \lambda_{A,1} + \epsilon^2 \sum_{k=1}^{N_B} (\mathbf{u}_{A,1}\mathbf{P}\mathbf{u}_{B,k}) a_k + o(\epsilon^3)\,, \tag{6}$$

$$\lambda_{T,2} = \lambda_{B,1} + \epsilon^2 \sum_{k=1}^{N_A} (\mathbf{u}_{B,1}\mathbf{P}\mathbf{u}_{A,k}) b_k + o(\epsilon^3)\,, \tag{7}$$

where $a_k = (\mathbf{u}_{A,1}\mathbf{P}\mathbf{u}_{B,k})/(\lambda_{A,1} - \lambda_{B,k})$, $b_k = (\mathbf{u}_{B,1}\mathbf{P}\mathbf{u}_{A,k})/(\lambda_{B,1} - \lambda_{A,k})$ and $\mathbf{P}$ is a matrix whose elements are $P_{lm} = P_{ml} = 1$ if nodes l of A and m of B are connected through a connector link, and $P_{lm} = P_{ml} = 0$ elsewhere.

With no loss of generality, we can neglect the $k > 1$ elements of the ϵ^2 terms in Eqs. (6-7) because they are far less relevant than the first element of the summation as $\lambda_{A,1} - \lambda_{B,k} > \lambda_{A,1} - \lambda_{B,1}$ for $k > 1$. Furthermore, we approximate the initial condition $\mathbf{n}(0)$ to

$$\mathbf{n}(\mathbf{0}) = C_A \mathbf{u}_{A,1} + C_B \mathbf{u}_{B,1}\,, \tag{8}$$

where $C_A^2 + C_B^2 = 1$, and $\mathbf{u}_{A,1}$ and $\mathbf{u}_{B,1}$ are as mentioned the first eigenvectors associated to networks A and B, meaning that initial populations on A and B before interconnecting both networks are already at equilibrium.

We introduce the distribution of population at time t between both networks $x(t)$ as

$$x(t) = \frac{\mathbf{n}(t) \cdot \mathbf{u}_{B,1}}{\mathbf{n}(t) \cdot \mathbf{u}_{A,1}}. \tag{9}$$

Note that (i) $x(t) \in [0, \infty]$, (ii) $x(t) \to 0$ when $\mathbf{n}(t) \to \mathbf{u}_{A,1}$ (that is, when most population is on network A), (iii) $x(t) = 1$ when $\mathbf{n}(t) \propto \mathbf{u}_{A,1} + \mathbf{u}_{B,1}$ (when the population is equally distributed on A and B), and (iv) $x(t) \to \infty$ when $\mathbf{n}(t) \to \mathbf{u}_{B,1}$ (when most population is on network B).

The evolution with time of the distribution of population $x(t)$ in the network of networks T, combining Eqs. (3) and (9), is given by

$$x(t) = \frac{\sum_{i=1}^{N_T} \lambda_{T,i}^t \alpha_i \mathbf{u}_{T,i} \cdot \mathbf{u}_{B,1}}{\sum_{i=1}^{N_T} \lambda_{T,i}^t \alpha_i \mathbf{u}_{T,i} \cdot \mathbf{u}_{A,1}}\,. \tag{10}$$

Dividing numerator and denominator in Eq. (10) by $\lambda_{T,1}^t$ and due to the exponentially fast suppression of the contributions due to higher-order terms (since $\lambda_{T,i} \geq \lambda_{T,i+1}$, $\forall i$), we obtain

$$x(t) \approx \frac{\mathbf{u}_{B,1} \cdot \mathbf{u}_{T,1} + \left(\frac{\lambda_{T,2}}{\lambda_{T,1}}\right)^t \frac{\alpha_2}{\alpha_1} \mathbf{u}_{B,1} \cdot \mathbf{u}_{T,2}}{\mathbf{u}_{A,1} \cdot \mathbf{u}_{T,1} + \left(\frac{\lambda_{T,2}}{\lambda_{T,1}}\right)^t \frac{\alpha_2}{\alpha_1} \mathbf{u}_{A,1} \cdot \mathbf{u}_{T,2}}\,. \tag{11}$$

Normalising Eqs. (4-5) such that $|\mathbf{u}_{T,1}| = |\mathbf{u}_{T,2}| = 1$, and introducing Eqs. (4), (5), (6) and (7) in Eq. (11), we finally obtain the temporal evolution of the system,

$$x(t) = \frac{K^t L + \frac{\epsilon F}{\Delta\lambda}}{1 - K^t L \frac{\epsilon F}{\Delta\lambda}} + \mathcal{O}(\epsilon^2)\,, \tag{12}$$

where

$$K = \left(\frac{\lambda_{B,1}\Delta\lambda - (\epsilon F)^2}{\lambda_{A,1}\Delta\lambda + (\epsilon F)^2}\right), L = \left(\frac{x_0\Delta\lambda - \epsilon F}{\Delta\lambda + x_0\epsilon F}\right),$$

$\Delta\lambda = \lambda_{A,1} - \lambda_{B,1}$, the initial population distribution $x_0 = \frac{\mathbf{n}(0)\cdot\mathbf{u}_{B,1}}{\mathbf{n}(0)\cdot\mathbf{u}_{A,1}} = \frac{C_B}{C_A}$, and

$$F = \mathbf{u}_{A,1}\mathbf{P}\mathbf{u}_{B,1} = \sum_{cl} \left(\mathbf{u}_{A,1}\right)_i \cdot \left(\mathbf{u}_{B,1}\right)_j\,, \tag{13}$$

being cl the set of connector links. Therefore, F represents the sum of the products of the eigenvector centralities of all connector nodes measured prior to interconnecting A to B, and for this reason we will name *connection strength* to ϵF and *normalised connection strength* to $\epsilon F/\Delta\lambda$. Note that peripheral-peripheral (PP) connections between networks lead to low values of F, while central-central (CC) connections (or a very large number of PP connections) lead to large values of F.

All $k > 1$ elements in the ϵ-terms of Eqs. (4-5), as well as all elements in their ϵ^2-terms, are not present in Eq. (12) because they are cancelled when being multiplied by $\mathbf{u}_{A,1}$ and $\mathbf{u}_{B,1}$. This fact leads to an expression for $x(t)$ that only depends on the first eigenvalues of the isolated networks $\lambda_{A,1}$ and $\lambda_{B,1}$, the centrality of their connector nodes (through F, that depends on the first eigenvectors $\mathbf{u}_{A,1}$ and $\mathbf{u}_{B,1}$), the value of the weight of the connector links ϵ and the initial condition (through x_0).

In summary, Eq. (12) describes the dynamics of the system for all times and can be used to obtain how much population will be at each network at any time, and how the system would evolve if we changed the connector links following any connection strategy.

In case we used a generic initial condition $\mathbf{n}(0)$ different to Eq. (8), the analytic expression becomes more troublesome during the first steps of the process, but in practice, after some steps, the population would spread over both networks and Eq. (12) would be fully applicable. See Supplementary Note 4 for details.

Supplementary Note 3. The population flux and the asymptotic equilibria

The population flux $\dot{x}(t)$ measures the speed at which the population of network A moves towards network B at time t. Making use of the expression for $x(t)$ at Eq. (11) we obtain

$$\begin{aligned}\dot{x}(t) &= \frac{dx(t)}{dt} \approx \left(\frac{\lambda_{T,2}}{\lambda_{T,1}}\right)^t \ln\left(\frac{\lambda_{T,1}}{\lambda_{T,2}}\right)\frac{\alpha_2}{\alpha_1} \\ &\times \frac{(\mathbf{u}_{B,1}\cdot\mathbf{u}_{T,1})(\mathbf{u}_{A,1}\cdot\mathbf{u}_{T,2}) - (\mathbf{u}_{A,1}\cdot\mathbf{u}_{T,1})(\mathbf{u}_{B,1}\cdot\mathbf{u}_{T,2})}{\left[(\mathbf{u}_{A,1}\cdot\mathbf{u}_{T,1}) + \left(\frac{\lambda_{T,2}}{\lambda_{T,1}}\right)^t \frac{\alpha_2}{\alpha_1}(\mathbf{u}_{A,1}\cdot\mathbf{u}_{T,2})\right]^2}\,,\end{aligned} \tag{14}$$

and introducing Eqs. (4), (5), (6) and (7) in Eq. (14) yields

$$\dot{x}(t) = K^t \ln(K)\, L \frac{1 + \left(\frac{\epsilon F}{\Delta\lambda}\right)^2}{\left(1 - K^t L \frac{\epsilon F}{\Delta\lambda}\right)^2} + \mathcal{O}(\epsilon^2)\,, \tag{15}$$

where K, L, $\Delta\lambda$, ϵ and F are as in Eq. (12).

$\dot{x}(t) = 0$ means that the system is in the equilibrium state. We can therefore calculate the asymptotic equilibria with an initial condition given by Eq. (8) equalising to zero the population flux shown in Eq. (14) (see Supplementary Note 7 for details): $\dot{x}(t) = 0$ for (i) $t \to \infty$ and (ii) $\alpha_2 = \mathbf{n}(0) \cdot \mathbf{u}_{T,2} = 0$. Equation (12) leads for the former that

$$x(t \to \infty) \to x_{\text{eq}} = \frac{\epsilon F}{\lambda_{A,1} - \lambda_{B,1}}\,, \tag{16}$$

while the latter implies that the only initial conditions that coincide with equilibrium states are also given by $x_{0,\text{eq}} = \epsilon F/(\lambda_{A,1} - \lambda_{B,1})$.

Supplementary Note 4. Population distribution and flux for arbitrary initial conditions

The dependence with time of the population distribution $x(t)$ and its flux $\dot{x}(t)$ on two interconnected networks is formally given by Eqs. (11) and (14) respectively, and they can be approximated by Eqs. (12) and (15) if the initial condition $\mathbf{n}(0)$ is $\mathbf{n}(0) = C_A \mathbf{u}_{A,1} + C_B \mathbf{u}_{B,1}$, where $C_A^2 + C_B^2 = 1$, meaning that initial populations on A and B before connection are already in equilibrium.

But what happens if we start the process with an arbitrary initial condition $\mathbf{n}(0)$? The only term of Eqs. (11-15) that depends on the initial condition is α_2/α_1,

$$\frac{\alpha_2}{\alpha_1} = \frac{\mathbf{n}(0) \cdot \mathbf{u}_{T,2}}{\mathbf{n}(0) \cdot \mathbf{u}_{T,1}}\,, \tag{17}$$

where $\mathbf{u}_{T,1}$ and $\mathbf{u}_{T,2}$ follow Eqs. (4) and (5). Substituting L by Eq. (17) in Eq. (12) for $x(t)$ and in Eq. (15) for $\dot{x}(t)$ allows to describe very precisely the evolution with time of the population distribution and its flux for all times and any initial condition $\mathbf{n}(0)$. The price to pay for this high precision is that these expressions depend on all the eigenvalues $\lambda_{A,i}$ and $\lambda_{B,i}$ and eigenvectors $\mathbf{u}_{A,i}$ and $\mathbf{u}_{B,i}$ of matrices associated to networks A and B respectively, while Eqs. (11-15) only depend on the first eigenvalues and eigenvectors of such matrices.

On the other hand, extensive numerical work shows that in most real cases the ϵ^2-terms in Eqs. (4-5) are negligible, and the same applies for the $k > 1$ elements of the ϵ-terms because they are far less relevant than the first element of the summation as $\lambda_{A,1} - \lambda_{B,k} > \lambda_{A,1} - \lambda_{B,1}$ for $k > 1$ in a_k, and $\lambda_{B,1} - \lambda_{A,k} > \lambda_{A,1} - \lambda_{B,1}$ for $k > 1$ in b_k for the relevant cases of $\lambda_{A,1} \approx \lambda_{B,1}$. After these approximations, we obtain

$$\frac{\alpha_2}{\alpha_1} = \frac{\mathbf{n}(0) \cdot \mathbf{u}_{T,2}}{\mathbf{n}(0) \cdot \mathbf{u}_{T,1}} \approx \frac{\mathbf{n}(0) \cdot \mathbf{u}_{B,1} + \epsilon b_1 \mathbf{u}_{A,1}}{\mathbf{n}(0) \cdot \mathbf{u}_{A,1} + \epsilon a_1 \mathbf{u}_{B,1}} = \frac{x_0 \Delta\lambda - \epsilon F}{\Delta\lambda + x_0 \epsilon F}\,. \tag{18}$$

Note that this expression is equal to the one obtained for networks A and B initially in equilibrium —L in Eqs. (12) and (15)—, although now the initial condition is arbitrary and $x_0 = \frac{\mathbf{n}(0)\cdot\mathbf{u}_{B,1}}{\mathbf{n}(0)\cdot\mathbf{u}_{A,1}}$ for any $\mathbf{n}(0)$. This result indicates that, while Eq. (12) for $x(t)$ and (15) for $\dot{x}(t)$ were obtained supposing populations on networks A and B initially at equilibrium, they are also applicable for most real and general cases after few time steps.

Supplementary Note 5. Generalisation to directed networks

When the transition matrix $\mathbf{M}$ is primitive and diagonalisable but not symmetric, that is, when the network under study is weighted and directed, the transition matrices $\mathbf{M_A}$, $\mathbf{M_B}$ and $\mathbf{M}$ are not symmetric. As a consequence, the left $\bar{u}_k^L$ and right $\bar{u}_l$ eigenvectors of these matrices do not coincide but are biorthonormal, that is, $\mathbf{u}_k^L \cdot \mathbf{u}_l = 1$, $\forall\ k = l$, and $\mathbf{u}_k^L \cdot \mathbf{u}_l = 0$, $\forall\ k \neq l$.

The formal expression of the dynamics depends on both the right and left eigenvectors of $\mathbf{M}$ such that

$$\mathbf{n}(t) = \mathbf{M}^t \mathbf{n}(0) = \sum_{i=1}^{m} \lambda_i^t \alpha_i^L \mathbf{u}_i \,, \tag{19}$$

where α_i^L is the projection of the initial condition $\mathbf{n}(0)$ on the $i-$th left eigenvector $\mathbf{u}_i^L$ of $\mathbf{M}$, $\alpha_i^L = \mathbf{n}(0)\,\mathbf{u}_i^L$. However, the asymptotic state of the population is again given by $\mathbf{u}_1$, which corresponds to the right eigenvector associated to the largest eigenvalue λ_1.

The calculations developed in the Results of the main manuscript and Supplementary Notes 2 and 3 should be conveniently modified for non-symmetric matrices taking into account that Eq. (19) depends on the left eigenvectors of the transition matrix. However, the results are formally very similar, making use of the expressions obtained in [8] for the eigenvectors and eigenvalues of two interconnected directed networks (in substitution of Eqs. (4-7)). As an example, Eqs. (4) and (6) become

$$\mathbf{u}_{T,1} = \mathbf{u}_{A,1} + \epsilon \sum_{k=1}^{N_B} a_k \mathbf{u}_{B,k} + \epsilon^2 \sum_{j=2}^{N_A} \sum_{k=1}^{N_B} \frac{a_k(\mathbf{u}_{A,j}^L \mathbf{P} \mathbf{u}_{B,k})}{\lambda_{A,1} - \lambda_{A,j}} \mathbf{u}_{A,j} + o(\epsilon^3) \,, \tag{20}$$

$$\lambda_{T,1} = \lambda_{A,1} + \epsilon^2 \sum_{k=1}^{N_B} (\mathbf{u}_{A,1}^L \mathbf{P} \mathbf{u}_{B,k}) a_k + o(\epsilon^3) \,, \tag{21}$$

where $a_k = (\mathbf{u}_{B,K}^L \mathbf{P} \mathbf{u}_{A,1})/(\lambda_{A,1} - \lambda_{B,k})$, ϵ is the weight of the connector links and $\mathbf{P}$ is the matrix that defines the connector links, and is not symmetric.

Note that to first order of ϵ only the connector links that go from the strong network to the weak network are relevant for the calculation of the eigenvector $\mathbf{u}_{T,1}$. Furthermore, in a general case where each connector link has a different weight (as it happens in the OECD Inter-Country Input-Output (ICIO) database studied in the main manuscript), the above equations show $\epsilon = 1$ and the weights of the links become the elements of matrix $\mathbf{P}$.

Finally, let us remark that as the Perron-Frobenius Theorem holds for any irreducible non-negative square matrix, our methodology can be applied to any network (directed or undirected) as far as its associated transition matrix is also irreducible. In practice, this happens when the graph is ergodic, that is, when any pair of nodes are connected through a sequence of links. If the matrix is not irreducible, it could happen that the system presented more than one asymptotic behaviour, and the phenomenology would be more complex than the one presented in our work.

Supplementary Note 6. Generalisation to more than 2 networks

The study of the out-of-equilibrium dynamics among $m > 2$ networks is a very rich phenomenon that deserves a detailed study beyond the scope of this work. However, we can present here a simplified example to show that the main highlights of the methodology presented in this paper are also applicable to the more complex system of m networks. Let us suppose that a network A is connected with a network BC that consists of two networks B and C joined by several connector links (that in this case can change

in time), and $\lambda_{A,1} > \lambda_{B,1} > \lambda_{C,1}$. The expressions for the eigenvector centrality and the first eigenvalue associated to network BC follow Eqs. (4) and (6):

$$\mathbf{u}_{BC,1} = \mathbf{u}_{B,1} + \epsilon \sum_{k=1}^{N_C} \frac{\mathbf{u}_{B,1}\mathbf{P}\mathbf{u}_{C,K}}{\lambda_{B,1} - \lambda_{C,k}} \cdot \mathbf{u}_{C,k} + o(\epsilon^2)\,, \quad (22)$$

$$\lambda_{BC,1} = \lambda_{B,1} + \epsilon^2 \sum_{k=1}^{N_C} \frac{(\mathbf{u}_{B,1}\mathbf{P}\mathbf{u}_{C,k})^2}{\lambda_{B,1} - \lambda_{C,k}} + o(\epsilon^3)\,. \quad (23)$$

We introduce the distribution of population at time t between network A and BC, $x_{A-BC}(t)$, as

$$x_{A-BC}(t) = \frac{\mathbf{n}(t) \cdot \mathbf{u}_{BC,1}}{\mathbf{n}(t) \cdot \mathbf{u}_{A,1}}. \quad (24)$$

If we study the connection of network A to network BC, in the way already shown in former calculations for 2 networks, we obtain the temporal evolution of the system,

$$x_{A-BC}(t) = \frac{K^t L + \frac{\epsilon_1 F_1}{\Delta\lambda}}{1 - K^t L \frac{\epsilon_1 F_1}{\Delta\lambda_1}} + \mathcal{O}(\epsilon^2)\,, \quad (25)$$

$$\dot{x}_{A-BC}(t) = K^t \ln(K)\, L \frac{1 + \left(\frac{\epsilon_1 F_1}{\Delta\lambda_1}\right)^2}{\left(1 - K^t L \frac{\epsilon_1 F_1}{\Delta\lambda_1}\right)^2} + \mathcal{O}(\epsilon^2)\,, \quad (26)$$

where

$$K = \left(\frac{\lambda_{B,1}\Delta\lambda_1 - (\epsilon_1 F_1)^2}{\lambda_{A,1}\Delta\lambda_1 + (\epsilon_1 F_1)^2}\right), L = \left(\frac{x_0 \Delta\lambda_1 - \epsilon_1 F_1}{\Delta\lambda_1 + x_0 \epsilon_1 F_1}\right),$$

$\Delta\lambda_1 = \lambda_{A,1} - \lambda_{BC,1}$, $\lambda_{BC,1} = \lambda_{B,1} + \epsilon_2^2 \frac{F_2^2}{\lambda_{B,1} - \lambda_{C,1}}$, the initial population distribution $x_0 = \frac{\mathbf{n}(0)\cdot\mathbf{u}_{BC,1}}{\mathbf{n}(0)\cdot\mathbf{u}_{A,1}} = \frac{C_{BC}}{C_A}$, and

$$F_1 = \mathbf{u}_{A,1}\mathbf{P}\mathbf{u}_{BC,1} = \sum_{cl} (\mathbf{u}_{A,1})_i \cdot (\mathbf{u}_{BC,1})_j\,, \quad (27)$$

$$F_2 = \mathbf{u}_{B,1}\mathbf{P}\mathbf{u}_{C,1} = \sum_{cl} (\mathbf{u}_{B,1})_i \cdot (\mathbf{u}_{C,1})_j\,, \quad (28)$$

being cl_1 the set of connector links that connect network A with networks B and C, and cl_2 the set of connector links that connect networks B and C.

The population flux diagram, in its simplest version, consists now of a 3-dimensional representation of the evolution with time of the distribution of population between network A and the rest of networks, $\dot{x}(t)$, where the X-axis represents $\epsilon_1 F_1/(\lambda_{A,1} - \lambda_{BC,1})$, where ϵ_1 and F_1 represent the weight and strength of the connector links that connect network A with the union of networks B and C, Y-axis represents $\epsilon_2 F_2(\lambda_{B,1} - \lambda_{C,1})$, where ϵ_2 and F_2 represent the weight and strength of the connector links that connect networks B and C, and Z-axis is $x(t)$, the evolution with time of the distribution of population between network A and the rest of networks.

A similar calculation would be derived if we focused on analysing the flux of centrality between networks A and B in the same 3-networked system. In this case, the temporal evolution of the system

would be given by the following expressions,

$$x_{A-B}(t) = \frac{\mathbf{n}(t)\cdot\mathbf{u}_{B,1}}{\mathbf{n}(t)\cdot\mathbf{u}_{A,1}} = \frac{x_{A-BC}(t)}{\sqrt{1+(\frac{\epsilon_2 F_2}{\lambda_{B,1}-\lambda_{C,1}})}}\,, \tag{29}$$

$$\dot{x}_{A-B}(t) = \frac{\dot{x}_{A-BC}(t)}{\sqrt{1+(\frac{\epsilon_2 F_2}{\lambda_{B,1}-\lambda_{C,1}})}}\,. \tag{30}$$

Supplementary Note 7. Equilibrium configurations

We equalise the population flux to zero to calculate the asymptotic equilibria of the system. Regarding Eq. (14), we obtain that $\dot{x} = 0$ for (i) $\lambda_{T,2} = 0$, (ii) $\lambda_{T,1} = \lambda_{T,2}$, (iii) $\alpha_2 = 0$ and (iv) $(\mathbf{u}_{B,1}\cdot\mathbf{u}_{T,1})(\mathbf{u}_{A,1}\cdot\mathbf{u}_{T,2}) = (\mathbf{u}_{A,1}\cdot\mathbf{u}_{T,1})(\mathbf{u}_{B,1}\cdot\mathbf{u}_{T,2})$. Note that the first case, $\lambda_{T,2} = 0$, is not possible in real networks, Perron-Frobenius theorem implies $\lambda_{T,1} > \lambda_{T,2}$, and $(\mathbf{u}_{B,1}\cdot\mathbf{u}_{T,1})(\mathbf{u}_{A,1}\cdot\mathbf{u}_{T,2}) < (\mathbf{u}_{A,1}\cdot\mathbf{u}_{T,1})(\mathbf{u}_{B,1}\cdot\mathbf{u}_{T,2})$ for all ϵ. Therefore, the only real equilibria are given by $\alpha_2 = 0$, which implies that an initial population distribution, given by $x_0 = (\mathbf{u}_{B,1}\cdot\mathbf{n}(0))/(\mathbf{u}_{A,1}\cdot\mathbf{n}(0))$, coincides with an equilibrium point if

$$x_{0,\mathrm{eq}} = \frac{\epsilon F}{\lambda_{A,1}-\lambda_{B,1}} + o(\epsilon^3)\,. \tag{31}$$

We observe here that $x_{0,\mathrm{eq}}$ grows indefinitely with the connection strength ϵF, but this behaviour has no physical meaning. Even when Eq. (31) allows for ϵF of any size, there is a maximum possible value of F in real systems (and in general $F << 1$), as it will represent the case in which both networks are connected through their most populated nodes (CC strategy). Also, the weight of the connector links ϵ should be small to make the Eqs. (4-7) applicable.

It is easy to see computationally that increasing indefinitely the weight of the links that connect two arbitrary real networks leads to an equivalent distribution of the population between both networks ($P_A = P_B = 0.5$, $x(t\to\infty) = 1$, see how the equilibrium lines in Fig. 2(a-b) and Fig. 3 of the main manuscript tend to a horizontal line for large $\epsilon F/(\lambda_{A,1}-\lambda_{B,1})$). Therefore, the system can never show an equilibrium in $x_{0,\mathrm{eq}} >> 1$, that is, when substantially more population remains on B than on A, which in our calculation leads to $\epsilon F/(\lambda_{A,1}-\lambda_{B,1}) \le 1$. That is the limit of applicability of Eqs. (12) for $x(t)$ and (15) for $\dot{x}(t)$, but it is safely beyond most practical cases.

In summary, the existence of an upper bound for the connection strength ϵF (and therefore for the normalised connection strength $\epsilon F/\Delta\lambda$) such that $x_{0,\mathrm{eq}}$ is maximum reveals that there are no equilibrium distributions very close to $\mathbf{u}_{B,1}$, while they can be as close to $\mathbf{u}_{A,1}$ as desired. This result coincides with what was already known: even in optimal connections for the weak network (central nodes of A connected to central nodes of B), the strong network obtains at equilibrium a large fraction of the total final population.

Supplementary Note 8. Alternating strategies

Our methodology can be applied in the context of game theory. From this viewpoint, the different networks compete for population following a certain amount of rules that should be fixed in each case. A simple example is that in which the networks alternate the choice of the connector links, trying to optimise their own population. We can solve analytically this situation, as follows.

With no loss of generality, we develop the population flux $\dot{x}(t)$ —see Eq. (15)— in series of ϵ and neglect the second order terms to simplify the calculations, obtaining

$$\dot{x}(t) = \left(\frac{\lambda_B}{\lambda_A}\right)^t \ln\left(\frac{\lambda_A}{\lambda_B}\right)\left(x_0 + x_0^2\left[2\left(\frac{\lambda_B}{\lambda_A}\right)^t - 1\right]\epsilon F\right) + o(\epsilon^2)\,. \tag{32}$$

In the equilibrium, the population flux provoked by one competitor must counteract the competing strategy, that is, $\dot{x}(t)' = -\dot{x}(t)''$, which yields

$$\begin{aligned}\dot{x}(t)' &= -\dot{x}(t)'' \Rightarrow \dot{x}(t)' + \dot{x}(t)'' = 0\\ &\Rightarrow \left(\frac{\lambda_B}{\lambda_A}\right)^t \ln\left(\frac{\lambda_A}{\lambda_B}\right)\left(x_0 + x_0^2\left[2\left(\frac{\lambda_B}{\lambda_A}\right)^t - 1\right]\frac{\epsilon' F' + \epsilon'' F''}{2}\right) + o(\epsilon^2) = 0\,. \end{aligned}\tag{33}$$

From here it is easy to see that the equilibrium distribution of alternating two different strategies coincides with that of applying both strategies simultaneously but with the link weights equal to the half of the original ones. Applying Eq. (31) we finally obtain that

$$x_{\mathrm{eq}}^{alter} \approx \frac{\epsilon' F' + \epsilon'' F''}{2(\lambda_{A,1} - \lambda_{B,1})} = \frac{x'_{\mathrm{eq}} + x''_{\mathrm{eq}}}{2}\,. \tag{34}$$

In summary, the distribution of population between both networks at the equilibrium due to alternating strategies is the average between the equilibria of each of the strategies applied independently. For example, if the strong network connects to the weak network through PP connections in the even turns, while the weak one connects to the strong one through CC connections in the odd turns, both trying to maximise their own final population, the equilibrium would in fact be an intermediate situation between what they would obtain if they were able to decide in all steps the best strategy for each of them.

Supplementary Note 9. Applicability of the framework

Our aim is to present a general methodology and a guide for its use, and therefore a complete analysis of a particular sample beyond the ones already studied in the main manuscript is far from the scope of this work. Anyway, we focus on several widely used mathematical models of complex systems and a very renowned network-based biotechnological dataset to remark the versatility of the methodology and help its applicability in different contexts.

Mathematical models

Our framework can be applied to the description of mutation-selection evolutionary processes. They model the evolution of heterogeneous populations (e.g. viruses or bacteria) on the space of genomes [9,10], where each node represents a genome and two nodes are connected if they differ in only one nucleotide. In fact, the space of genomes has been recently proved to behave as a network of networks [9,11]. In this context the transition matrix becomes

$$\mathbf{M} = R(1-\mu)\mathbf{I} + \frac{R\mu}{S}\mathbf{G}\,, \tag{35}$$

where R is the replication rate, $\mu < 1$ is the probability of mutating from one genome to another, $\mathbf{I}$ is the identity matrix, and S stands for the maximum number of neighbours of a node. A different field of application could be the control and optimisation of the growth and spreading of species on fragmented habitats, where Eq. (35) is also valid being μ the probability per unit time of an organism moving from one region to another.

We can also focus on disease spreading in social environments. For instance, the Susceptible-Infected (SI) model can be described by a transition matrix of the form $\mathbf{M} = \beta\mathbf{G}$, and the Susceptible-Infected-Recovered (SIR) model $\mathbf{M} = \beta\mathbf{G} - \gamma\mathbf{I}$, where β and γ are the probabilities per unit time that an healthy individual will be infected or an infected individual will recover, respectively (see [12], for details of the two different models). In these two cases, $n_i(t)$ represents the probability of individual i of being infective at time t. Note that in both models there are only two potential final states, the infection or recovery of the whole population. This fact yields that our methodology will be applicable if (i) the parameters place the system close to the epidemic threshold (where the infected population remains bounded because the largest eigenvalue of matrix $\mathbf{M}$ verifies $\lambda_1 = \gamma/\beta$ for the SIR model), or (ii) for short times, before the whole population has either been infected or totally recovered.

Finally, the diffusion of rumours is strongly related to epidemic processes. The Maki-Thompson (MK) model [13] considers three different kinds of actors in a population: ignorants (who never heard about the rumour), spreaders (who spread the rumour to their contacts) and stiflers (who have heard the rumour but decide to stop its diffusion). One ignorant becomes a spreader of the rumour with a probability α and when two spreaders meet, one of them realises that the rumour does not have novelty and becomes a stifler with a probability β. The dynamics of the fraction of spreaders in the MK model can be described by a transition matrix of the form $\mathbf{M} = (\alpha + \beta)\mathbf{G} - \beta\mathbf{I}$, similar expression to that of the SIR model.

Real datasets

A fruitful application of our methodology to real data associated to networked systems could be the analysis of an extensive 12-year (1988-1999) monitoring of the evolution of the structure and dynamics of interorganisational collaboration among almost three thousand biotechnological institutions [14]. This massive study optimally fulfils the conditions to be analysed with the methodology here presented, as (i) the networks of collaboration in biotechnology are composed of many inter-connected subnetworks [15], (ii) they are permanently out of equilibrium because they are characterised by a high rate of creation and destruction of connections, as entities that do not either expand or renew their networks finally lose their central positions, and (iii) the nodes with the most central positions in the field are not the firms with larger market power or more capability of creating novelty, but those with a more diverse group of well-connected collaborators [14], supporting eigenvector centrality as the ideal quantity to measure the importance of an organism in this kind of networks.

References

1. Coleman, J.S., Katz, E. & Menzel, H. The diffusion of an innovation among physicians. *Sociometry* **20**, 253-270 (1957).

2. Coleman, J.S., Katz, E. & Menzel, H. *Medical Innovation: A Diffusion Study.* Indianapolis: Bobbs-Merrill (1966).

3. König, M.D., Battiston, S., Napoletano, M. & Schweitzer, F. On algebraic graph theory and the dynamics of innovation networks. *Netw. Heterog. Media* **3**, 201–219 (2008).

4. König, M.D. & Battiston, S. *From Graph Theory to Models of Economic Networks. A Tutorial.* Springer Berlin Heidelberg, Berlin, Heidelberg, 23–63 (2009).

5. König, M.D., Battiston, S., Napoletano, M. & Schweitzer, F. Recombinant knowledge and the evolution of innovation networks. *J. Econ. Behav. Organ.* **3**, 145–164 (2011).

6. Banerjee, A., Chandrasekhar, A.G., Duflo, E. & Jackson, M.O. The diffusion of microfinance. *Science* **341**, 1236498 (2013).

7. OECD, Inter-Country Input-Output (ICIO) Tables, 2018 edition. Available at http://www.oecd.org/sti/ind/inter-country-input-output-tables.htm

8. Aguirre, J., Papo, D. & Buldú, J.M. Successful strategies for competing networks. *Nat. Phys.* **9**, 230 (2013).

9. Aguirre, J., Catalán, P., Cuesta, J.A. & Manrubia, S. On the networked architecture of genotype spaces and its critical effects on molecular evolution. *Open Biol.* **8**, 180069 (2018).

10. Aguirre J, Buldú JM, Manrubia SC (2009) Evolutionary dynamics on networks of selectively neutral genotypes: Effects of topology and sequence stability. *Phys. Rev. E* **80**, 066112.

11. Yubero, P., Manrubia, S. & Aguirre, J. The space of genotypes is a network of networks: implications for evolutionary and extinction dynamics. *Sci. Rep.* **7**, 13813 (2017).

12. Newman, M.E.J. *Networks: An introduction.* Oxford University Press, New York (2010).

13. Maki, D.P. & Thompson N. *Mathematical models and applications, with emphasis on social, life, and management sciences.* Prentice Hall, Englewood Cliffs, NJ (1973).

14. Powell, W.W., White, D.R., Koput, K.W. & Owen-Smith, J. Network dynamics and field evolution: The growth of interorganizational collaboration in the life sciences. *Am. J. Sociol.* **110**, 1132–1205 (2005).

15. Orsenigo, L., Pammolli, F. & Riccaboni, M. Technological change and network dynamics: lessons from the pharmaceutical industry. *Res. Policy* **30**, 485–508 (1996).
