# Supplementary material for: Taming out-of-equilibrium dynamics on interconnected networks
Source: Nat Commun. 2019 Nov 22;10:5314. doi: 10.1038/s41467-019-13291-2 (PMC6874616; doi:10.1038/s41467-019-13291-2)
Supplement: Supplementary file 2 — Description of Additional Supplementary Files [file 41467_2019_13291_MOESM2_ESM.pdf]

Description of Additional Supplementary Files

Supplementary Movie 1.

Animated description of the normalised connection strength for every pair of OECD countries in the period (2005-2015). As the normalised connection strength between two networks/countries A and B is given by the expression $(\vec{u}^{L}_{B,1}\mathbf{P}\vec{u}_{A,1})/(\lambda_{A,1}-\lambda_{B,1})$, where $\lambda_{A,1} > \lambda_{B,1}$, the matrix is symmetric. Also, note that while the economic relation between some countries and the rest remains similar during the whole period (e.g. Japan), others drastically changed their connections after the 2008 crisis (e.g. Iceland). Data obtained from reference [7] of the Supplementary Information.
